# Columnar Mesophases and Organogels Formed by H-Bound Dimers Based on 3,6-Terminally Difunctionalized Triphenylenes

**DOI:** 10.3390/gels11010009

**Published:** 2024-12-27

**Authors:** Nahir Vadra, Lisandro J. Giovanetti, Pablo H. Di Chenna, Fabio D. Cukiernik

**Affiliations:** 1 Departamento de Química Inorgánica, Analítica y Química Física, Facultad de Ciencias Exactas y Naturales, Universidad de Buenos Aires, Buenos Aires C1428EGA, Argentina; vadra@qi.fcen.uba.ar; 2 Instituto de Química Física de los Materiales, Medio Ambiente y Energía (INQUIMAE), Universidad de Buenos Aires, CONICET, Buenos Aires C1428EGA, Argentina; 3 Instituto de Investigaciones Fisicoquímicas Teóricas y Aplicadas (INIFTA), Facultad de Ciencias Exactas, Universidad Nacional de la Plata, CONICET, La Plata B1900, Argentina; lisandro@fisica.unlp.edu.ar; 4 Unidad de Microanálisis y Métodos Físicos Aplicados a la Química Orgánica (UMYMFOR), Departamento de Química Orgánica, Facultad de Ciencias Exactas y Naturales, Universidad de Buenos Aires, CONICET, Buenos Aires C1428EGA, Argentina

**Keywords:** triphenylene functionalized triphenylenes, columnar liquid crystals, organogel, H-bonded dimers

## Abstract

A series of triphenylene (TP) compounds—denoted 3,6-THTP-DiC*n*OH—bearing four hexyloxy ancillary chains and two variable-length alkoxy chains terminally functionalized with hydroxyl groups have been synthesized and characterized. The shorter homologs revealed mesogenic characteristics, giving rise to thermotropic mesophases in which π-stacked columns of H-bound dimers self-organize yielding superstructures. Molecular-scale models are proposed to account for their structural features. The three studied compounds yielded supramolecular gels in methanol; their ability to gelify higher alcohols was found to be enhanced by the presence of water. The intermediate homolog also gelled *n*-hexane. Compared to their isomeric 2,7-THTP-DiC*n*OH analogs, the 3,6-derivatives showed a higher tendency to give rise to LC phases (wider thermal ranges) and a lower organogelling ability (variety of gelled solvents, lower gels stabilities). The overall results are analyzed in terms of different kinds of competing H-bonds: intramolecular, face-to-face dimeric, lateral polymeric, and solvent–TP interactions.

## 1. Introduction

The way different components in soft materials self-organize at a molecular level is the result of a balance between different non-covalent interactions and the disorder induced either by temperature or by the presence of solvent. It can thus be expected, in principle, that substances that tend to organize into soft phases in the bulk—like mesogens giving rise to liquid crystal (LC) phases [1]—will also exhibit some degree of propensity to self-organize in the presence of a solvent—like, for example, low molecular weight organogelators (LMWGs) [2,3]. Many mesogenic substances have been shown to be efficient LMWGs [4,5,6,7], involving a wide pallet of non-covalent interactions [8,9,10,11]. However, the number of substances studied both as mesogens and organogelators is still limited, and their design remains a challenge. Although the term “organogelling liquid crystals” was coined more than 15 years ago [12], the structural details associated to the way such species self-organize in LC phases or in gels can be markedly different; the comparison between these two aspects remains incipient [13,14,15,16].

**Figure 1:**
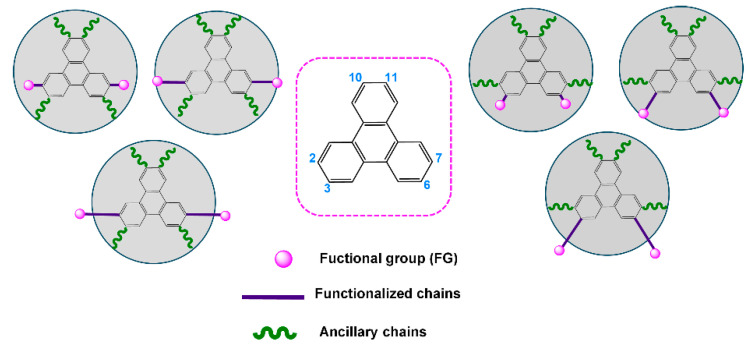
General description of triphenylenes based on a three-block molecular architecture, terminally functionalized at different positions ((**left**): 2,7; (**right**): 3,6). Pink: functional group; violet: functional chains (alkoxy chain bearing FG at the free end); green: ancillary chains. The schematic representation emphasizes the position of the FG relative to the “crown” (grey circle) formed by the disordered ancillary chains, (**center**): numbering scheme.

Triphenylenes have been widely studied as building units for soft materials, such as liquid crystals [17,18,19], gels [14,20,21], thin films [22], polymers [23,24,25], etc. They exhibit interesting molecular and self-assembling properties, whose potential increases when they are based on a multi-block molecular architecture [26,27,28]. Indeed, the presence of terminal functional groups (FGs) at the free end of some of the aliphatic chains surrounding the core of 2,3,6,7,10,11-hexa(alkoxy)triphenylenes (Figure 1) provides, on the one hand, the key for their incorporation into macromolecular systems, like linear [29,30], branched [31,32], or cross-linked polymers [33]; on the other hand, it allows for the development of intermolecular interactions (FG–FG, FG–solvent, FG–support), eventually leading to specific materials, such as LC [17,18], LB films [34], gels [14,19,35], etc. However, synthetic aspects associated with this molecular architecture often become a barrier for the preparation of multi-block triphenylene-based novel materials. Among other strategies developed to overcome this difficulty, recent suggestions of simple and robust synthetic pathways for some key precursors [36,37,38] contributed to paving the way.

In this context, we recently studied [39] both the mesogenic and organogelling ability of hexa(alkoxy)triphenylenes bearing four hexyloxy ancillary chains at the 3,6,10,11 positions and two variable-length alkoxy chains terminally functionalized with hydroxyl groups at the 2 and 7 positions. Those compounds, schematically represented in Figure 1 (left), were denoted 2,7-THTP-DiCnOH (THTP = tetra(hexyloxy)triphenylene).

Despite the extensive work carried out during the last 30 years concerning mesogenic triphenylenes, the influence of the diverse molecular blocks on the structure and stability of their condensed phases remains a matter of very active research [40,41,42]. Different molecular parameters can exert a decisive influence on the properties of the described three-block materials. On one hand, the effect of the length of the functionalized chains—or the relative length of the functionalized vs. the ancillary chains—on the LC properties of monomers [43,44], oligomers [45] and polymers [46] has been explored; however, studies of this parameter on the organogelling properties still remain scarce. On the other hand, the functionalization pattern has been found to play a very important role in the LC properties of polymers [47] and LB films [34], although it has been suggested [34] that its influence on the LC properties is secondary (but see below). Finally, the nature of the FG proved to have a strong influence on both the LC and organogelling properties [20,39,44].

Among different possible FGs, hydroxyl is particularly interesting: not only it can give rise to different kind of co-polymers, but it is also a very efficient H-bond maker, acting both as donor and as acceptor. Our recent study on the 2,7-THTP-DiCnOH series [39] showed those compounds exhibited thermotropic mesomorphism and were able to act as supramolecular gelators in a variety of alcohols. Their gels were remarkably stable in methanol, and their stability was increased by the presence of water in alcoholic mixtures. The analogous tetra(pentyloxy)triphenylenes substituted at the 2,3- and 3,6-positions, 2,3-TPTP-DiCnOH and 3,6-TPTP-DiCnOH, respectively, have been reported previously, but their phases have not been fully characterized: some mesophases were characterized as unknown LC phases or unidentified Discotic phases, as they were not analyzed by X-ray diffraction (XRD) to determine their nature, but only by polarized optical microscopy (POM) and differential scanning calorimetry (DSC).

**Figure 2:**
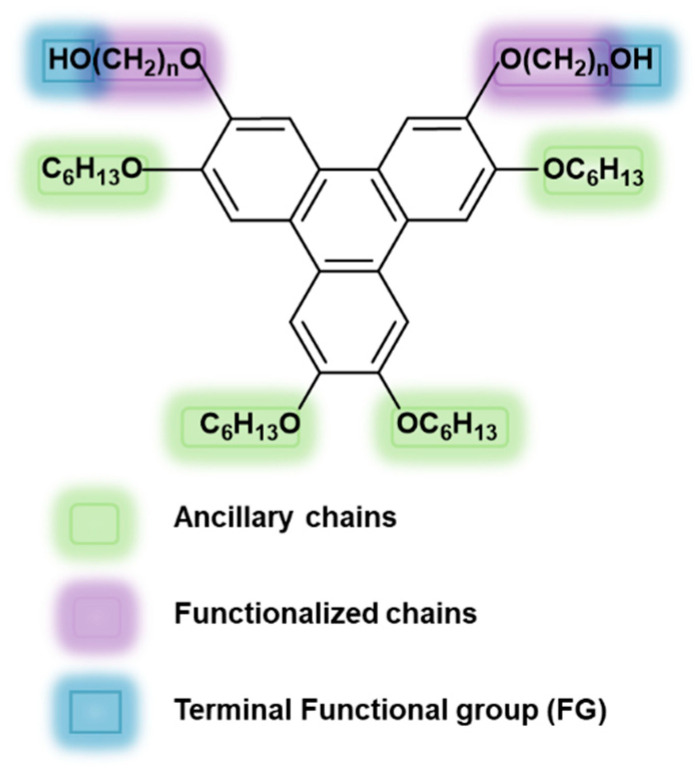
Compounds studied in this work.

In the present study, we analyze the organizing properties of TPs terminally functionalized with hydroxyls in the alkoxy chains located at positions 3,6 of a hexa(alkoxy)triphenylene bearing four linear hexyloxy chains at the other positions (as depicted in Figure 1 (right)). To compare the results with the previously reported 2,7-isomers, we analyze a series of three compounds in which the terminal hydroxyl groups are attached to the TP core at the 3,6-positions by alkoxy chains of varying lengths, and the other four positions (2,7,10,11-) are occupied by hexyloxy chains (Figure 2): 3,6-THTP-DiC*n*OH. We seek for the influence on both the mesomorphic and organogelling properties of the functionalization pattern as well as relative length of the functionalized vs. ancillary chains. We present here these results, and interpret them in terms of molecular-scale models for the supramolecular organization in both the bulk mesophases and the gels. The analysis of the whole set of results for the different di-OH functionalized TP-series allows for a general picture about the way H-bond interactions develop in each case to give rise to LC mesophases and organogels.

## 2. Results and Discussion

### 2.1. Synthesis and Characterization

**Scheme 1:**
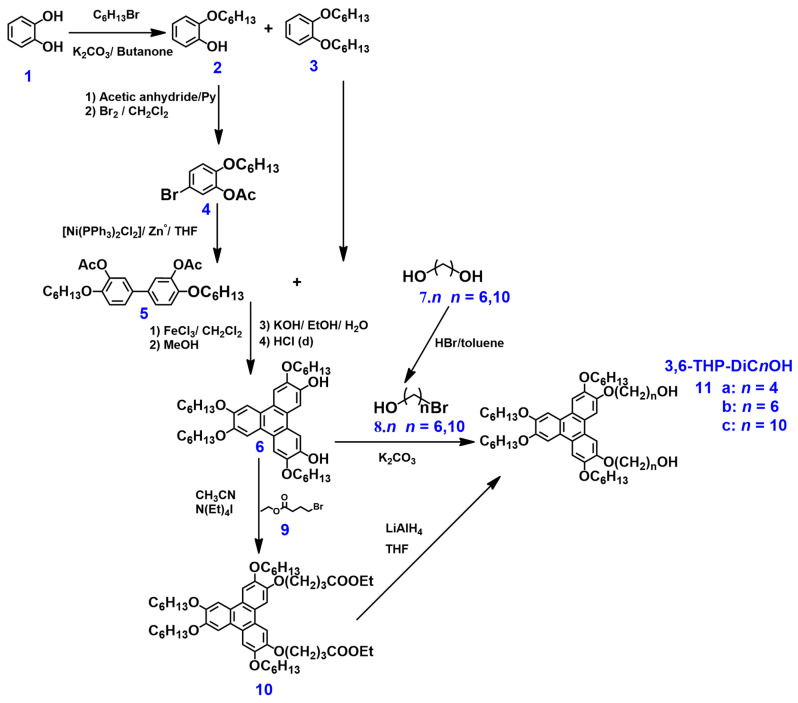
Synthetic strategy used to obtain the 3,6-THT-DiCnOH; *n* = 4, 6 and 10.

A di-phenolic triphenylene precursor bearing two OH groups in positions 3 and 6 (3,6-THTP-DiOH) was synthesized, in the framework of the “rational synthesis” approach, according to Scheme 1. This synthetic pathway is similar to the one already used for the synthesis of 2,7-THTP-DiOH [39], but the order of the acetylation/bromination steps was inverted, leading to the targeted 3,6-isomer. Indeed, as bromination of activated aromatic compounds using molecular bromine as reagent lead to substitution in ortho- or para- positions relative to the activating group, direct bromination of **2** will yield a mixture of isomers. For that reason, a key step in the synthesis of 3,6-THTP-DiOH precursor **6** was acetylation of the phenolic group of **2**: as hexyloxy chains are stronger activants than acetoxy groups, bromination was mainly directed to the para- position with respect to the hexyloxy group, yielding compound **4**, the appropriate isomer to be further transformed into 3,6-THTP-DiOH. Selective bromination was then performed under strict control of temperature (lower than 5 °C), reaction time, and Br_2_ addition speed, in order to minimize the extent of polybromination; nevertheless, the required purification steps lead to overall yields lower than those obtained for the 2,7-THTP-DiOH analogue [36,39].

The next two steps (Ullman’s coupling using a nickel catalyst in the presence of powdered Zn, and an oxidative coupling of the acetylated biphenyl with di(hexyloxy)benzene in the presence of ferric chloride) required a strict inert atmosphere and, thus, were performed using either Schlenck techniques or a glovebox. This synthetic sequence yielded a mixture of the expected precursor **6** and its acetylated derivative, which was subsequently subjected to basic hydrolysis. After chromatographic purification in the dark, the 3,6-THTP-DiOH precursor **6** was isolated in low overall yield. The targeted 3,6-THTP-DiC6OH and 3,6-THTP-DiC10OH compounds have been obtained through direct Williamson’s etherification of **6** with the corresponding α,ω-bromoalcohol. For the lightest homologue, we were unable to purify the intermediate compound **8** (*n* = 4) and then decided to follow an alternative synthetic pathway [39], involving etherification with a commercial bromo-ester (**9**) followed by its reduction to the targeted 3,6-THTP-DiC4OH derivative.

Compounds **4**, **5**, and **6** were characterized by NMR techniques (see experimental section and Figure S1). The nature and purity of the targeted final 3,6-THTP-DiC*n*OH compounds (*n* = 4, 6, 10) have been established based on their ^1^H-NMR and ^13^C-NMR spectra (see experimental section for details and Figures S2 and S3). Their ^1^H-NMR spectra exhibited the multiplets corresponding to methyl and methylene groups in the 0–2 ppm region, the triplets corresponding to methylene α- to the terminal hydroxyl groups at ca. 3.7–3.8 ppm, and those corresponding to CH_2_ α- to the ether linkage at ca. 4.25 ppm. The last signal appears split for the shortest *n* = 4 homologue, where the proximity of the terminal OH groups to the aromatic core slightly reduces the overall symmetry, giving rise to distinctive signals for the OH terminally functionalized chains; their relative integrations support this interpretation. On the other hand, the aromatic protons gave rise to a single signal for the three compounds of this series, owing to their higher symmetry compared to that of **6**. The ^13^C-NMR spectra of the 3,6-THTP-DiC*n*OH compounds showed three signals (six for the lighter and less symmetric homologue) corresponding to aromatic C-atoms in the 105–150 ppm region, with one peak at 63 ppm corresponding to CH_2_ groups α-to terminal hydroxyl groups, one peak (two for the *n* = 4 derivative) at *ca* 70 ppm corresponding to the methylene C atoms α- to the ether linkages, a series of signals in the 20–35 ppm region corresponding to the other methylene groups, and a single peak at 14 ppm assigned to terminal methyl groups (Figure S2).

### 2.2. Mesomorphic Properties

**Figure 3:**
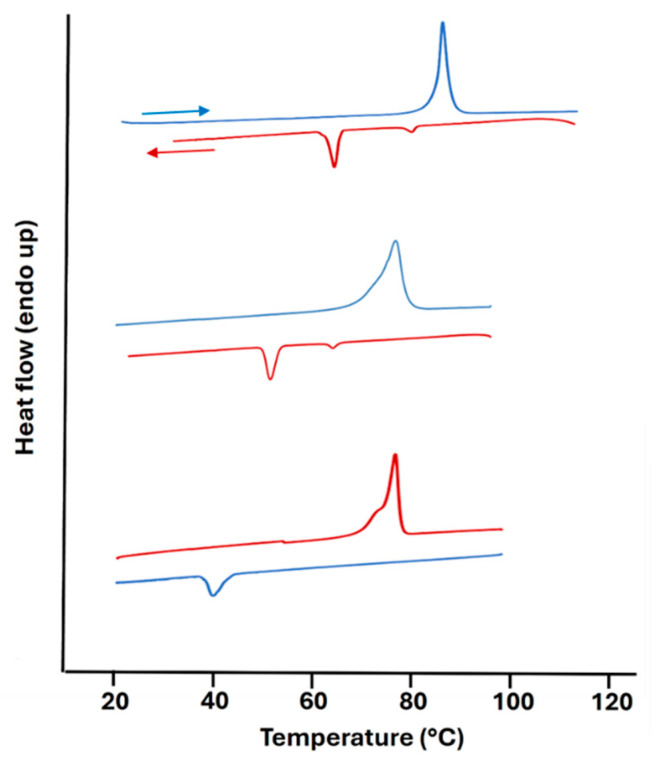
DSC traces of the second heating–cooling cycles for 3,6-THTP-DiC*n*OH (top: *n* = 4, middle: *n* = 6, bottom: *n* = 10).

**Figure 4:**
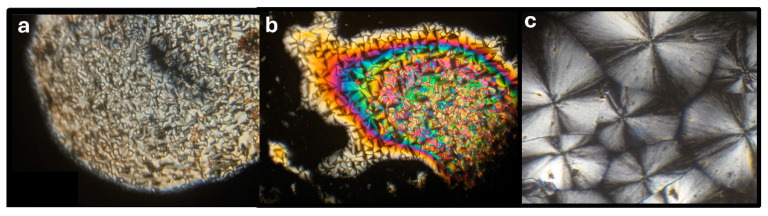
POM pictures taken on cooling from the isotropic state for the studied 3,6-THTP-DiC*n*OH compounds: (**a**): *n* = 4 at 78 °C, (**b**): *n* = 6 at 62 °C, (**c**): *n* = 10 at room temperature.

**Figure 5:**
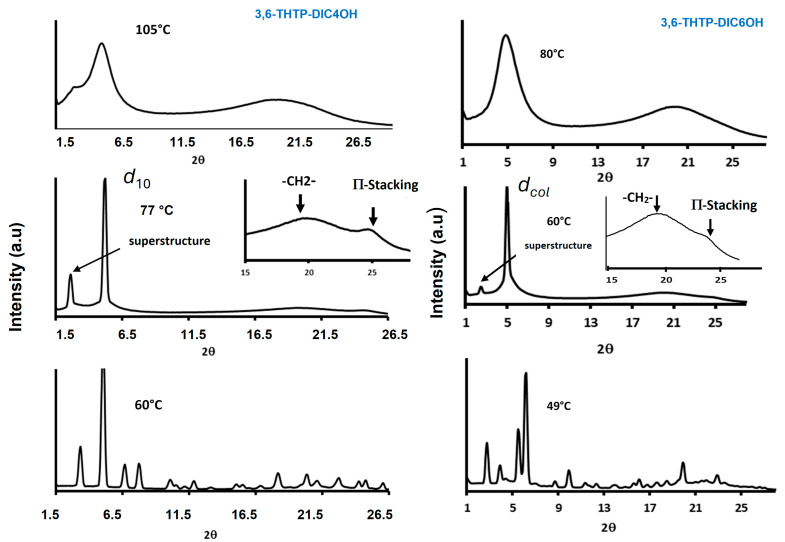
XRD patterns for 3,6-THTP-DiC4OH (**left**) and 3,6-THTP-DiC6OH (**right**) at selected temperatures.

The mesomorphic properties of the three compounds under investigation were examined using polarized optical microscopy (POM), differential scanning calorimetry (DSC), and variable-temperature X-ray diffraction (XRD) through small-angle X-ray scattering/wide-angle X-ray scattering (SAXS/WAXS). These studies are summarized in Figure 3, Figure 4 and Figure 5; thermal and structural details are given on Table 1. As can be seen, both the 3,6-THTP-DiC4OH and 3,6-THTP-DiC6OH compounds exhibited monotropic columnar mesophases, whereas the heavier 3,6-THP-DiC10OH homolog proved to be non-mesogenic. DSC traces of the two lightest homologs showed one endothermic peak on heating and two exothermic peaks on cooling. POM observations in the temperature range between the two exothermic peaks detected on cooling are typical of columnar mesophases, in agreement with XRD patterns, which exhibit one narrow peak near 2*θ* = 5° associated with intercolumnar distances (*d_col_*), a wide halo at ca. 2*θ* = 19° typical of molten aliphatic chains, and a peak at 2*θ* = 25° assigned to intra-columnar *π*−stacking distances. Both compounds exhibited an additional weak peak at small angles, assigned to a superstructure, as discussed below. A full list of the peaks detected in the diffractograms of n = 4 and 6 compounds in both the solid and LC phases is given in Table S1; an additional comparison is performed in Figure S4.

**Figure 6:**
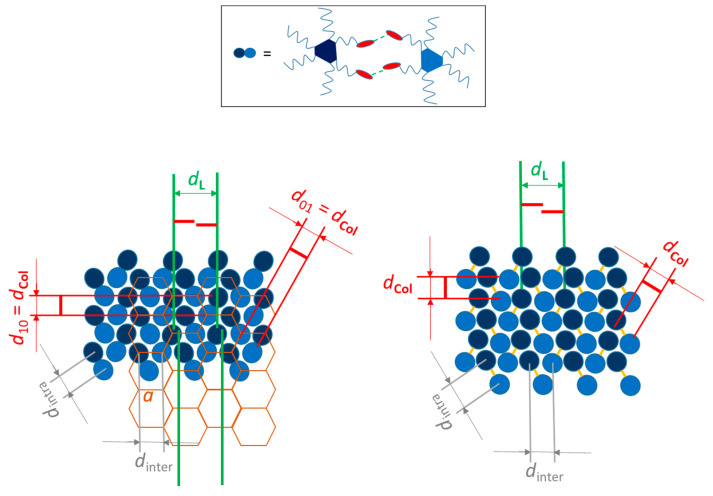
Proposed models for the organization of the triphenylene molecules in the columnar mesophase of 3,6-THTP-DiC4OH (**left**) and 3,6-THTP-DiC6OH (**right**), explaining their main structural features including the lamellar superstructure. **Top**: schematic representation of the suggested H-bond dimers.

The most intense peak in the diffraction patterns of the Col mesophases found for 3,6-THTP-DiC4OH and 3,6-THTP-DiC6OH, labeled *d*_col_, is certainly associated to the intercolumnar arrangement of the stacked triphenylene units; in a strict hexagonal array of identical columns with intercolumnar distance *a*, it corresponds to the 10 or 01 planes (with *d*_10_ = *d*_01_ = 0.5√3.*a*). The presence of a weak peak (labeled *d*_L_) associated with a distance twice that of *d*_col_ for 3,6-THTP-DiC4OH points to the presence of some kind of superstructure, in which vicinal columns are no longer identical, although similar. This feature can easily be understood when looking at the molecular structure of the studied compounds, where H-bound dimers are likely expected. This kind of arrangement, depicted in Figure 6 (left), gives rise to molecules differing just in orientation, in such a way that the electronic density along the material varies essentially with a periodicity compatible with one molecular diameter, but is slightly modulated by the dimeric association, thus yielding a superstructure characterized by a structural parameter (*d*_L_) twice that associated to the molecular diameter (*d*_10_ = *d_Col_* = 0.5√3*a*), as shown in Figure 6 (left). This kind of organization is known in the CLC field as “lamello-columnar mesophases” Col_L_ or LamCol [48]. The fact that *d*_L_ = 2*d*_Col_ requires the interdimeric distance (*d*_inter_) to be identical to the intradimeric one (*d*_intra_). If the intradimeric distance is not the same as the interdimeric one (e.g., for longer bridging chains), then *d*_L_ is not expected to be twice *d*_Col_ (the system is not strictly hexagonal). As depicted in Figure 6 (right), a modulation of the electronic density following the molecular diameter is still expected, but *d*_L_ may now differ from 2*d*_Col_; indeed, for intradimeric distances slightly longer than interdimeric ones, *d*_L_ < 2*d*_Col_ could be expected, as depicted in Figure 6 (right). It is worth noting that although the preceding analysis has been presented in terms of molecular and not columnar arrangements, it could be expected that the H-bonds scheme involves molecules at different planes, in such a way that the “molecules” depicted in Figure 6 represent a normal section of columns locating the H-bonds in the same intercolumnar region.

### 2.3. Organogelating Ability

**Figure 7:**
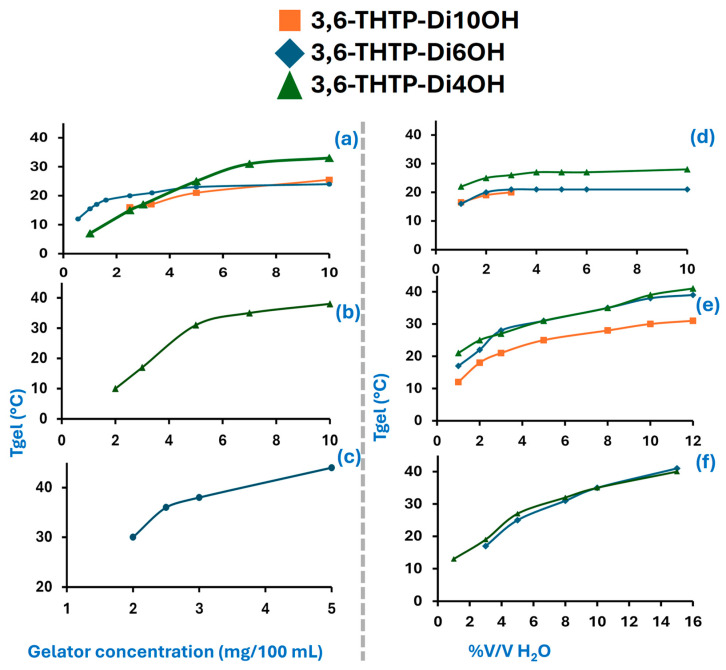
(**Left**) Plot of Tgel as a function of 3,6-THTP-DC*n*OH concentration in (**a**) methanol, (**b**) ethanol, and (**c**) n-hexane. (**Right**) Plot of Tgel as a function of %V/V water added in mixtures of (**d**) methanol, (**e**), ethanol and (**f**) 2-propanol.

The organogelating properties of the three compounds were investigated using the inverted tube method (see the experimental section for details); the results are summarized in Table 2. The gel-to-sol transition temperature (T_gel_) vs. concentration dependence was also explored, exhibiting the expected behavior for supramolecular gels, i.e., T_gel_ increasing with concentration up to a plateau zone where a maximum T_gel_ (T_gel_^max^) is reached (Figure 7 (left)). The three compounds were able to form supramolecular gels in methanol (see Figure S5), although with different stability levels. The compound containing the longest functionalized chains, 3,6-THTP-DiC10OH, exhibited the highest critical gelation concentration and the lowest thermo-stability compared to the shorter chain analogs. In the presence of less polar solvents, such as ethanol, only the shortest functional chain compound, 3,6-THTP-DiC4OH, formed stable gels up to 38 °C (Figure 7b). In contrast to what was previously observed with the 2,7-isomers [39], the 3,6-isomers were not able to form stable supramolecular structures with alcohols that were longer than two carbon atoms. However, the compound 3,6-THTP-DiC6OH has the unique ability, compared to shorter or longer functional chain compounds and compared to 2,7 isomers, to exhibit organogelation in a non-polar solvent, such as *n*-hexane (Figure 7c)

In our previous work on the 2,7-isomers [39], the sensitivity of the thermostability of these gels to the presence of small amounts of water allowed us to construct curves as a useful semiquantitative method to evaluate the water content in some light alcohols. With this idea in mind, we proceeded to evaluate the sensitivity of the gels formed by the 3,6-isomers to small additions of water.

Despite the absence of gel formation in alcohols other than methanol (except for *n* = 4), the addition of small amounts of water enabled the formation of stable gels in ethanol and 2-propanol. Table 3 illustrates the minimum percentage by volume (%V/V) of water (detection limit) necessary for the formation of a stable gel, while Figure 7 (right) shows the stability curves constructed for the compound–alcohol–water systems that exhibited stable gel formation. A distinctive feature of these systems, in contrast to those with position 2,7 analogs, was the formation of stable gels in methanol–water systems. Although consecutive additions of small percentages of water in methanol generated stable gels, they did not show variability in the T_gel_ as a function of the % of addition. These systems do not allow a qualitative measurement of the water content by observing the T_gel_. In particular, the compound 3,6-THTP-DiC10OH showed a very narrow range of stability.

As mentioned above, although no stable gels were formed with 3,6-THT-DiC10OH and 3,6-THT-DiC6OH in ethanol, the addition of small amounts of water generated the formation of stable gels, as shown in Figure 7d. It was observed that an increase in the length of the functionalized chain decreases the thermal stability of these gels, reflected in a lower T_gel_ value.

The selectivity of these compounds for the detection of small amounts of water in alcohol was only possible for 2-propanol–water mixtures in a range of 1–12%V/V for 3,6-THT-DiC4OH and 3,6 THT-DiC6OH. However, in ethanol–water mixtures within the aforementioned range, all three compounds were able to form stable gels with temperature sensitivity as a function of % water.

**Figure 8:**
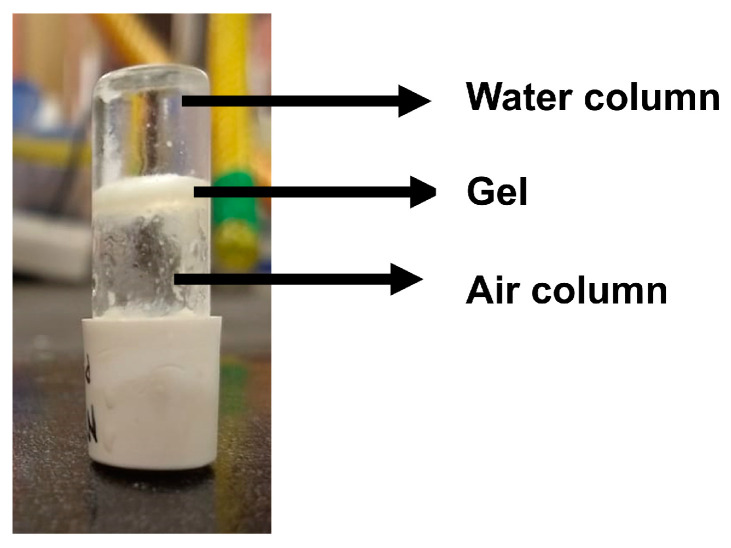
Photograph of inverted tube with gel formed from 3,6THTP-DIC6OH in water–hexane at a ratio of 80:20.

The presence of water in heterogeneous systems, such as hexane–water, did not result in any modification of the behavior of the supramolecular gel in terms of T_gel_ and minimum gelation concentration. Upon the addition of a specified quantity of 3,6-THT-DiC6OH to a hexane–water mixture, gelation of the organic phase was observed following a heating, stirring, and cooling process, without the formation of a precipitate or the rupture of the gel. In heterogeneous systems comprising predominantly water (80:20 water–hexane systems), the water column is observed in the lower portion (Figure 8). Moreover, when the tube is inverted, the gel demonstrates sufficient stability to support the water column’s weight.

**Figure 9:**
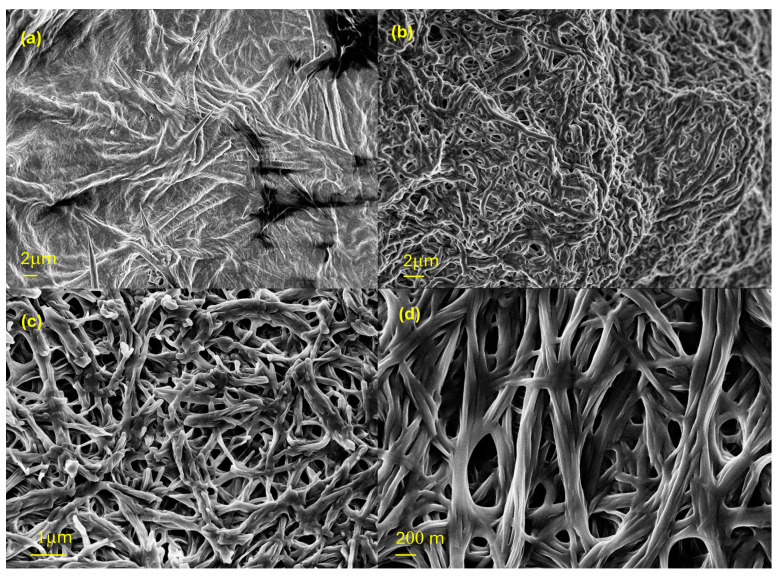
SEM images of 3,6-THT-DiC*n*OH xerogels obtained from methanol. (**a**) *n* = 10, (**b**) *n* = 6, and (**c**) *n* = 4, and 3,6-THT-DiC6OH in hexane (**d**).

To obtain an insight into the morphology of the self-assembled supramolecular structures of the organogels, we also performed SEM microscopy of the xerogels. High-vacuum drying provoked the collapse of the microstructure of the systems, so we slowly evaporated the solvent at room temperature and atmospheric pressure and the solids obtained were metalized with a film of platinum before analysis. In all xerogels, the images showed the presence of crossed-linked fibrillar networks (Figure 9), which is the typical morphology observed for xerogels of low molecular weight organogels, suggesting the presence of a self-assembled fibrillar network in the gel phase.

### 2.4. Supramolecular Models for the Columnar Mesophases and Organogels

As stated above, in the thermotropic mesophases of both 3,6-THTP-DiC4OH and 3,6-THTP-DiC6OH, the columns of stacked triphenylene molecules self-organize within an array characterized by a *d*_Col_ structural parameter; in turn, the asymmetric nature of the molecular units and the formation of H-bound dimers give rise to a higher-order lamellar superstructure, characterized by a d**_L_** structural parameter, yielding an overall LamCol mesophase. The difference between the superstructures observed for 3,6-THT-DiC4OH and 3,6-THT-DiC6OH resides in the relative values of *d***_Col_** and *d***_L_**. According to the proposed supramolecular dimer model, the difference could be ascribed to the fact that for *n* = 6 the intradimer core–core separation is larger than the interdimeric one (*d*_intra_ > *d*_inter_, Figure 6); in contrast, for the *n* = 4 homologue, the distances would be almost identical (*d*_intra_ = *d_i_*_nter_ Figure 6). The reasons leading to one or the other situation for each of these two compounds are analyzed in the following paragraphs in more detail.

**Figure 10:**
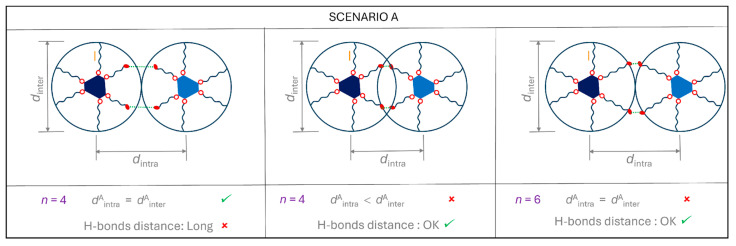
Analysis of the structural features expected for supramolecular dimers of 3,6-THTP-DiC4OH and 3,6-THTP-DiC6OH under the assumption of extended zig-zag aliphatic chains.

**Figure 11:**
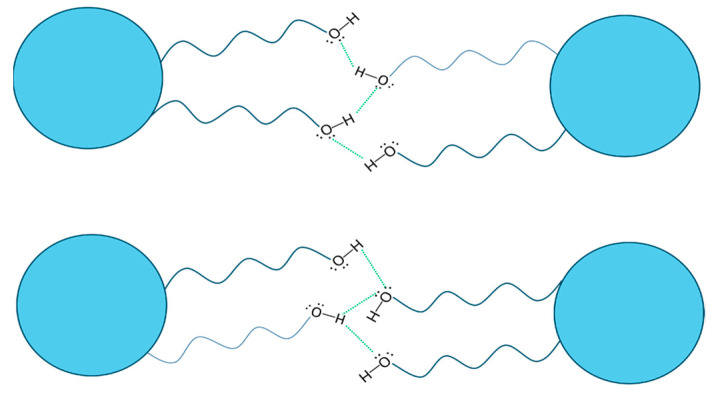
Two possible interaction schemes involving multiple H-bonds (green doted lines) between 3,6-THTP-DiC*n*OH molecules giving rise to supramolecular dimers.

Figure 10 shows three hypothetical situations in the framework of an idealized model (which we called “scenario A”) in which the aliphatic chains radially depart from the core in an extended zig-zag-*trans* conformation, giving rise to “discs” with diameters which are the sum of that of the triphenylene’s core and the aliphatic chains’ extension. For *n* = 4, if the disks were “in contact”, the condition *d*_intra_ = *d*_inter_ would be fulfilled, but the OH groups would be located at exceedingly long distances to build up H-bonds (Figure 10 left). The formation of such H-bonds would be possible if the two molecules giving rise to the supramolecular dimer approached each other (Figure 10 (middle)). In such a case, however, the condition *d*_intra_ = *d*_inter_ will not be fulfilled. For *n* = 6, the relative extension of both lateral and peripheric chains looks optimal to yield H-bonds; however, the subjacent condition *d*_intra_ = *d*_inter_ for “disks in contact” disagreed with the experimental structural results for this compound (Figure 10—right). In other words, none of the idealized situations analysed under the framework of “Scenario A” offer an explanation of the structural results. However, there is neither reason to assume aliphatic chains will adopt a full zig-zag trans conformation nor to limit the intermolecular interaction to just one pair of H-bonds taking place between two aliphatic chains. Actually, the relative orientation of the 3,6-functionalized chains allows the occurrence of multiple connectivity schemes, as usually found in H-bound supramolecular systems, due to the synergistic feature of H-bond interactions. Figure 11 schematically depicts two possible interaction schemes between the hydroxyl groups of the lateral chains of a supramolecular dimer.

**Figure 12:**
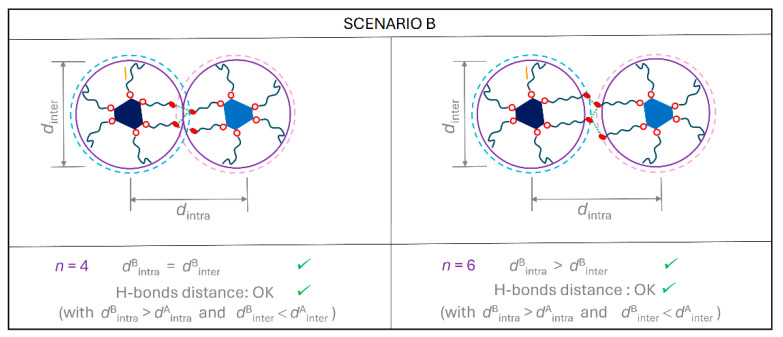
Schematic representation of a model for the LC phase that includes the multiple H-bonds schemes, a higher concentration of the functionalized chains in the inner region of the supramolecular dimers, and “molten” ancillary chains, which agrees with all the experimental facts.

If the strict condition of “fully extended aliphatic chains” is relaxed, a different picture arises. The “multiple H-bonds scheme” depicted in Figure 11 concentrates methylene groups in the region between the two TP cores of a dimer; maintaining the global density requires the peripheral aliphatic chains to fold to fill the space. This picture of molten aliphatic chains is indeed a widely accepted one for most columnar mesophases. Figure 12 shows the way that this hypothesis (mostly extended terminal aliphatic chains along the inter-core axis, multiple H-bonds, molten peripheral aliphatic chains; scenario B) allows us to explain the structural features of the superstructures of the Col_L_ mesophases of both the 3,6-THTP-DiC4OH and 3,6-THTP-DiC6OH compounds.

Although the previous discussion has been presented in terms of H-bound discrete dimers, the multiple H-bonds scheme can also include connections between TP molecules belonging to different dimers: the main features of the model will be maintained (the higher density of extended aliphatic chains in the region between the TP cores, molten aliphatic chains in the surrounding region), but the columnar character will be reinforced by “interplane” H-bond interactions.

**Figure 13:**
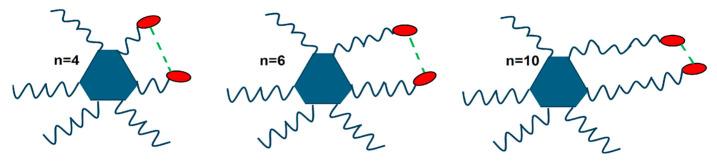
Schematic model depicting the increasing feasibility of intramolecular H-bonds (green doted lines) as the functionalized chains increase in length, thus destabilizing the supramolecular gels formed by 3,6-THT-DiC*n*OH and alcohols. The red ovals represent OH groups.

As far as the organogelling properties of the studied compounds are concerned, the observed tendency in the gelation ability with polar solvents can be attributed to the existence of a competition between the intramolecular hydrogen bond capacity between the terminal functional group (-OH) of each molecule and the hydrogen bonding interactions between neighboring molecules, as well as between the compound and the solvent. The experimental evidence suggests that, as the functionalized chain length increases, intramolecular hydrogen bond interactions become more feasible, resulting in a loss of stability in the supramolecular systems that are formed (Figure 13). This also explains why the compound 3,6-THT-DiC4OH is the only one capable of forming stable gels with a less polar alcohol, such as ethanol.

This analysis is also in accordance with the results obtained in alcohol–water mixtures. Although no gel formation was detected in alcohols higher than methanol (except for *n* = 4), all three compounds gelled mixtures of ethanol–water, and only 3,6-THTP-DiC4OH and 3,6-THTP-DiC6OH gellified 2-propanol–water mixtures. This behavior provides further confirmation for the proposed model. The addition of water to alcohols serves to increase the polarity of the solvent system, thereby overcoming intramolecular interactions that would otherwise impede gel formation. In the less polar solvents, such as 2-propanol–water, this effect can only be achieved by compounds with a reduced intramolecular hydrogen bond capacity, specifically 3,6-THTP-DIC4OH and 3,6-THTP-DiC6OH.

### 2.5. Comparison with Related Compounds: Influence of the Molecular Blocks on Self-Organizing Ability

**Figure 14:**
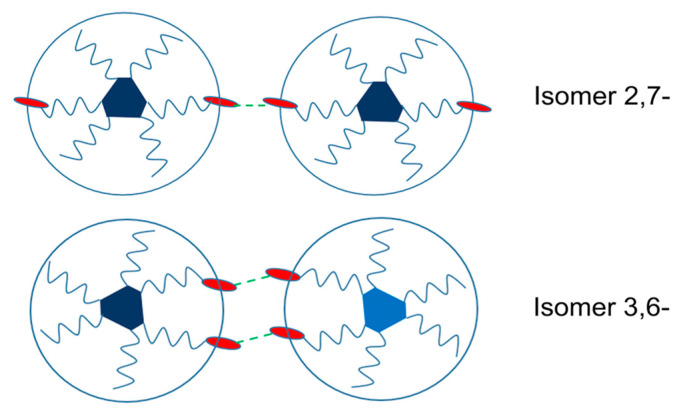
Schematic representation of the hydrogen bond interactions in the 2,7- and 3,6-functionalized isomers (extended lateral *vs.* localized). Red ovals represent OH groups, dotted green lines represent hydrogen bonds.

The results obtained previously on the 2,7-isomers showed the strong dependence of the organization properties on the relative length of the ancillary chains and the functionalized chains. This ratio governed the ability of these molecular units to form hydrogen bonds both between TP molecules as well as with different solvents. When the lengths of the functionalized chains and the ancillary chains were the same, lateral hydrogen bonding interactions were optimized, resulting in the formation of nematic mesophases rather than columnar ones, and in the formation of more stable gels with polar solvents. In addition to being influenced by the relative length of the chains, the self-assembly of the 3,6-isomers studied in the present work exhibited an additional modulation as a result of the potential these systems exhibit for the occurrence of other H-bond schemes, involving intramolecular ones (Figure 13), as well as multiply-bound dimers (Figure 14) or even columns.

The influence of the relative length of the functionalized vs. the ancillary chains on the mesomorphic properties of both the 3,6-THTP-DiC*n*OH and 2,7-THTP-DiC*n*OH series (*n* = 4, 6, 10) is in line with partial results obtained by Heiney and coworkers on the mesomorphic properties of 3,6-TPTP-DiC*n*OH and 2,3-TPTP-DiC*n*OH (TPTP = tetra(pentyloxy)triphenylene, *n* = 2, 3, 6) compounds [34], as well as with those reported by Shimizu et al. for the mesogenic behaviour of 2,3-THTP-DiC*n*CO_2_H (n = 3, 4, 5) [43,49] and by Wan et al. for a 2,7-TPTPDiOH and 2,7-TPTPDiC*n*CO_2_H (*n* = 3, 4, 5) series [44]. Although the characterization of the LC phases has been carried out in all these cases, albeit only by DSC and/or POM in [43], the general trends in stability shows that compounds in which the FGs at the end of the functionalized chains are at a radial distance appropriate for both H-bond interactions and close disk–disk contacts exhibit the more stable or organized LC phases, as we inferred for the 2,7- and 3,6-THTP-DiC*n*OH series on the basis of POM, DSC, and SAXS/WAXS experiments. The presence of H-bound dimers giving rise to superstructures has also been pointed out by Shimizu et al. based on IR studies [49].

Heiney et al. suggested [34] that although the relative length of the functionalized vs. the ancillary chains is a key factor controlling their mesomorphic properties, the substitution pattern is not. This suggestion, based on the comparison of only 2,3- and 3,6-isomers, disagrees with our findings on the 2,7- vs. 3,6-isomeric series. Indeed, the 2,7-THT-DiC6OH compound exhibited just an N_D_ mesophase, which was much less organized than the Col**_L_** exhibited by its 3,6-isomer. This different behavior can certainly be ascribed to the very different H-bond schemes expected for both compounds, namely lateral hydrogen bond interactions for the 2,7-isomer (Figure 14 top) vs. multiple H-bond schemes for the 3,6-derivative (Figure 14 bottom). This analysis also allows us to explain the lack of differences between the mesomorphic behavior of the 2,3- and 3,6-series stated by Heiney et al. [34] (both 3,6- and 2,3-isomers are well suited for multiple H-bond schemes) as well as the slight difference in the “optimal” relative length of the functionalized vs. the ancillary chains found for 2,7- vs. 2,3- or 3,6-systems. Indeed, in both the 2,3- and 3,6-isomers the multiple H-bonds scheme increases the aliphatic chains density in the inter-core region (thus maintaining a high “effective length”), forcing the ancillary chains to fold, leading to a lower “effective length” or disk diameter, yielding more stable/organized mesophases. As stated above, the multiple H-bonds scheme could also include interactions between molecules belonging to successive dimers, thus reinforcing the columnar character of the mesophase, in line with the Col mesophases found for these compounds vs. the ND mesophases found for the 2,7-analogs.

The functionalization pattern also defines the organogelling ability of these kinds of compounds, an unexplored feature up to now. The lower availability of the hydroxyl groups to interact with polar solvents explains the decreased gel-forming ability and gel stability for the 3,6 isomers as compared to the 2,7-derivatives. This difference arises from the ability of the 3,6-derivatives to build up either supramolecular dimers or higher nuclearity entities with multiple H-bond schemes (*n* = 4, 6) or intramolecular H-bonds (*n* = 10). This effect can be overcome by increasing the polarity of the solvents used (alcohol–water mixtures). However, this lower availability is reflected in systems with lower sensitivity for the detection of small amounts of water in alcohols.

## 3. Conclusions

The synthesized hexa-substituted triphenylenes bearing two hydroxyl groups at terminal positions of the 3,6-positions, 3,6-THTP-DiC*n*OH, exhibited both mesogenic columnar characteristics and organogelling ability. This series allowed for a comparison of the mesomorphic properties of the whole set of 2,3-, 3,6-, and 2,7-THTP-DiC*n*OH derivatives, as well as with the related TPTP analogs. The key role of the relative length of the functionalized and ancillary chains was, thus, confirmed for the whole set of isomers; the influence of the functionalization pattern on the kind and stability of the mesophases was established. In addition to the capability of such triphenylenes to self-organize in bulk mesophases and as LB films, their organogelling ability has been firmly established. Organogelling properties were also found to depend on both the relative lengths of ancillary and functionalized chains and the functionalization pattern. Not only the stabilities of the gels were sensitive to this last parameter. Moreover, each of the two series exhibited a distinctive unique feature: the 2,7-isomers showed the ability to detect the presence of small amounts of water in solvents, such as ethanol and 2-propanol, and even to quantify it within certain ranges; the 3,6-isomer bearing functionalized and ancillary chains of similar length (*n* = 6) had the unique ability to gel in non-polar solvents, such as *n*-hexane.

The whole set of results, involving both LC and organogelling properties for the different studied series, has successfully been interpreted in terms of the ability of these molecular units to give rise either to intramolecular H-bond interactions, simple or multiple intermolecular H-bond interactions (building up supramolecular dimers or oligomers), or intermolecular H-bonds with appropriate solvents, like alcohols.

## 4. Materials and Methods

Physicochemical measurements: Mesomorphic properties were studied by means of variable temperature polarizing optical microscopy (POM), differential scanning calorimetry (DSC), and variable temperature wide and small angle X-ray scattering (WAXS and SAXS). Elemental analyses and DSC experiments were carried out at INQUIMAE, on a Carlo Erba CHNS-O EA1108 analyzer (Emmendingen, Germany) and a Shimadzu DSC50 calorimeter (Kyoto, Japan), respectively. ^1^H and ^13^C-NMR spectra were measured on a Bruker Avance Neo 500 spectrometer (Fällanden, Switzerland), using CDCl_3_ as solvent and its residual peaks as internal references (7.26 ppm for ^1^H and 77.0 ppm for ^13^C). Mass spectra were recorded at CIBION in a Xevo G2S QT (Milford, UK) with ESI ion source. POM was carried out between crossed polarizers using a Leitz DMRX microscope (Wetzlar, Germany) equipped with a Leitz 1350 hotstage. SAXS/WAXS experiments were performed using a XEUSS 1.0 (XENOCS, Grenoble, France) with a Cu Ka; radiation parallel X-ray beam microsource. A Plilatus 100K was employed with a 533 cm sample–detector distance for SAXS geometry and a distance of 10 cm for WAXS experiments. Samples were placed in borosilicate capillaries and inside a temperature-controlled sample-holder from Linkam^®^ (Salfords, UK). Each sample was kept at a temperature above the isotropic phase transition for at least 30 min before cooling down with a 1 °C/min cooling rate. The gelation ability was investigated by a typical inverted tube experiment. A mixture of a defined amount of gelator and a volume of the solvent (10% wt./v) in a closed flask was heated and shaken until the solid was dissolved and then cooled down to 0 °C. If a stable gel was observed after inversion of the flask, it was considered a gel (G). The critical concentration for gelation (CCG) was determined by subsequent dilution of the original organogel followed by a heating–cooling process until gel formation was not observed. SEM pictures were taken on a Carl Zeiss NTS SUPRA 40 FEG scattering electron microscope (Oberkochen, Germany). The xerogels were prepared by slow evaporation of the solvent at room temperature and coated with a thin layer of platinum prior to examination.

Synthesis: The commercial reagents used were catechol, bromohexane, 1-6-hexanediol, 1-10-decanediol, Zn (<10 μm), FeCl_3_, ethyl 4-bromobutanoate, and LiAlH_4_, purchased from Sigma Aldrich or Fluka. Compounds **2**, **3**, **4**, and **5** and the complex NiCl_2_[P(Ph)_3_)]_2_ were synthesized following previously reported procedures [38,50]. The synthesis of **7** (*n* = 6 and 10) followed protocols described in [39].

**3,6-THTP-DiOH (6)**

Firstly, 0.910 g (1.9 mmol) of compound **5** and 2.602 g (9.3 mmol) of compound **3** were placed in a Schlenk’s flask. Three cycles of Ar/vacuum of 30 min each were carried out. Subsequently, anhydrous CH_2_Cl_2_ (30 mL) was added, and the flask was cooled in an ice bath. Then, 8.809 g of FeCl_3_ was added slowly under argon flow, the flask was covered, and the mixture was stirred for 20 min. The mixture was then allowed to return to room temperature. Following a four-hour reaction period, the flask was cooled in an ice bath, and 35 mL of cold methanol were added. The mixture was then left in a refrigerator overnight. The precipitate formed was filtered and washed with portions of cold methanol to obtain a green solid. The solid was introduced to a flask containing 35 mL of ethanol, 4 mL of water, and 1 g of KOH. The system was purged with argon and heated, maintaining reflux for a period of three hours. Subsequently, the content was transferred to a 50 g ice bath containing 5 mL of HCl (c), resulting in the precipitation of a white-greenish solid. Then, 25 mL of CH_2_Cl_2_ were added, and the aqueous phase was extracted on two occasions with 10 mL portions of CH_2_Cl_2_ each. The organic phase was dried with anhydrous Na_2_SO_4_, filtered, and evaporated under reduced pressure, yielding a violet solid. The product was purified by column chromatography (cyclohexane: CH_2_Cl_2_ 2:8) and subsequent recrystallization in 2-propanol, yielding 0.318 g of a white solid (R: 25%). ^1^H-NMR (500 MHz, CDCl_3_) δ (ppm): 7.96 (s, 2H); 7.84 (s, 2H); 7.78 (s, 2H); 5.89 (s, 2H); 4.31 (t, J = 6,5 Hz, 4H); 4.25 (t, J = 6.5 Hz, 4H); 2.02–1.9 (m, 8H); 1.59–1.53 (m, 8H); 1.47–1.41 (m, 16H); 0.96–0.93 (m, 12H).

**3,6-THTP-DiCOOEt (10)**

Firstly, 0.201 g (0.3 mmol) of 3,6-THTP-DiOH (**6**) were placed in a Schlenk’s flask. Three cycles of vacuum/argon were performed. Subsequently, under argon flow, 15 mL of anhydrous CH_3_CN, 0.021 g of tetraethyl ammonium iodide, and 0.620 g (4.3 mmol) of dry K_2_CO_3_ were added. The mixture was then heated for 30 min. Then, 0.20 mL (1.5 mmol) of ethyl 4-bromobutanoate **9** dissolved in 1 mL of CH_3_CN was added and heated again at reflux for 24 hr. Then, a portion of CHCl_3_ was added to the mixture, filtered, and the solution was evaporated. The product was purified by recrystallization with 2-propanol yielding 0.210 g of a beige solid (R = 79%). ^1^H-NMR (δ ppm): 7.93 (s, 2H); 7.86 (s, 2H); 7.85 (s, 2H); 4.33 (t, J = 6.2 Hz, 4H); 4.25 (m, 4H); 4.19 (q, J = 7.1 Hz, 4H); 2.67 (t, J = 7.3 Hz, 4H); 2.28 (m, 4H); 2.02–1.90 (m, 8H); 1.66–1.52 (m, 8H); 1.47–1.39 (m, 16H); 1.33–1.26 (m, 10H); 1.23 (t, J = 6.9 Hz, 6H); 0,96 (t, J = 6.9 Hz, 12H).

**3,6-THTP-DiC4OH (11a):**

A solution of 0.210 g of compound **10** (0.22 mmol) in 10 mL of anhydrous tetrahydrofuran (THF) was prepared in a Schlenk flask. The system was purged with argon, the flask was placed in an ice bath, and a solution of 0.120 g of LiAlH_4_ (2.6 mmol) in 2 mL of anhydrous THF was added. Following the addition, the ice bath was removed, and the reaction was stirred at room temperature for four hours. Subsequently, 5 mL of cold methanol was added, and the solution was extracted with two portions of CH_2_Cl_2_; the organic phase was dried with anhydrous Na_2_SO_4_, filtered, and evaporated under reduced pressure. The resulting solid was recrystallized in n-heptane, yielding 0.161 g of a white solid (R: 90%). ^1^H-NMR (δ ppm): 7.86 (s, 2H); 7.85 (s, 2H); 7.84 (s, 2H); 4.32 (t, J = 5.9 Hz, 4H); 4.26 (t, J = 6.6 Hz, 8H); 3.83 (t, J = 6.1 Hz, 4H); 2.26 (s, 2H); 2.13–2.07 (m, 4H); 2.02–1.94 (m, 12H); 1.38–1.36 (m, 24H); 0.99–0.93 (m, 12H).

^13^C-NMR (δ ppm): 149.07; 148.75; 148.38; 123.66; 123.63; 123.33; 107.43, 106.71; 106.40 (C-aromatics); 69.78; 69.37; 62.36 (-O-CH_2_); 31.70; 31.67; 30.01; 29.44; 29.29; 25.93; 25.87; 25.85; 22.68(-CH_2_-); 14.07(-CH_3_).

**3,6-THTP-DiC6OH (11b):**

Firstly, 0.160 g (0.24 mmol) of compound **6** (3,6-THT-DiOH) were placed in 10 mL of methyl ethyl ketone in a Schlenk’s flask. Subsequently, 1 g of K_2_CO_3_ and 0.184 g (1 mmol) of 6-bromo-1-hexanol were added to the mixture. The mixture was maintained at reflux under an argon atmosphere for 48 h. Subsequently 5 mL of water was added, and the solution was extracted with two 10 mL portions of CH_2_Cl_2_. The organic phase was washed with 1M HCl, dried with anhydrous Na_2_SO_4_, filtered, and evaporated under reduced pressure. The reaction product was purified by column chromatography (cyclohexane–ethyl acetate 70:30) and subsequent recrystallization with n-heptane to give 0.165g of a beige solid (R: 80%). ^1^H-NMR (δ ppm): 7.86 (s, 6H); 4.31–4.2 (m, 12H); 3.73 (t, J = 6.5 Hz, 4H); 1.97–1.94 (m, 12H); 1.72–1.63 (m, 20H); 1.48–1.35 (m, 16H), 0.96 (t, J = 6.8 Hz, 12H). ^13^C-NMR (δ ppm): 149.01; 148.97; 23.69, 123.61; 107.45; 107.36; 107.28 (C-aromatics); 69.73; 69.67; 69.59; 62.93; (O-CH_2_-); 32.74; 31.68; 29.44; 29.40; 26; 25.86; 25.61; 22.67 (-CH_2_); 14.07 (-CH_3_).

**3,6-THTP-DIC10OH (11c)**

The procedure is analogous to that previously described for 11b, with the exception of the addition of 0.114 g (0.16 mmol) of compound 6 and 0.151 g (0.6 mmol) of 10-bromo-1-decanol. The product was recrystallized from n-heptane and then methanol, resulting in 0.106 g of a beige solid (R: 69%). ^1^H-NMR (δ ppm): 7.86 (s, 6H); 4.25 (t, J = 6.6 Hz, 12 H); 3.66 (t, J = 6.6 Hz, 4H); 2.01–1.91 (m, 12H); 1.64–1.63 (m, 20H); 1.46–1.38 (m, 32H); 0.96 (t, J = 6.9 Hz, 12H) ^13^C-NMR (δ ppm): 149.99; 123.60; 107.36; (C-aromatics); 69.73; 63.08 (-O-CH_2_-); 32.82; 31.70; 29.59; 29.47; 29.44; 26.18; 25.85; 25.76; 22.68 (-CH_2_-); 14.07 (-CH_3_).

## Figures and Tables

**Table 1:** Phase sequence on cooling for the three studied compounds (transition temperatures in °C; transition enthalpies between parentheses in kJ mol^−1^) and structural characterization of the LC phases. The LC parameter has been calculated as $a=\sqrt{\frac{4}{3}}d_{Col}$ which is strictly right for the hexagonal system (*n* = 4) but an approximation for the other one (*n* = 6).

Compound	Phase Sequence	T (°C)	Dexp. (Å)	d_hk_	Lattice Parameter (Å)
3,6-THTP-DiC4OH	**I** 81 (4) **Col_h_** 66 (40) **Cr**	77	16.95 33.83	*d_10_* _=_ *d_col_* *d_L_* = 2*d_10_*	19.57
3,6-THTP-DiC6OH	**I** 65 (5) **Col_h_** 52 (63) **Cr**	60	18.02 35.32	*d_col_* *d_L_* < 2*d_col_*	20.80
3,6-THTP-DiC10OH	**I** 45 (83) **Cr**	-	-	-	-

**Table 2:** Gelation inverted tube test results: after a heating–cooling process the system was classified as follows: S: soluble; TG: turbid gel, and I: insoluble. The initial concentration was 10% wt./V. Critical concentration for gelation (CCG) values are shown between brackets (% wt./V).

*3,6-THTP-DiCnOH*			
Solvent	*n* = 4	*n* = 6	*n* = 10
Methanol	TG (1%)	TG (0.5%)	TG (2%)
Ethanol	TG (2%)	S	S
2-Propanol	S	S	S
n-Butanol	S	S	I
Acetonitrile	S	S	I
1,4-butanediol	I	I	I
Pentanol	S	S	S
n-hexane	I	TG (2%)	I
n-heptane	I	I	I
cyclohexane	S	S	S
Pentane	I	I	S
Dichloromethane	S	S	S
Acetone	S	S	S
Toluene	S	S	S
Ethyl acetate	S	S	S
Ethylene glycol	I	I	I

**Table 3:** Detection limit of water in light alcohols.

*3,6-THTP-DiCnOH*		
*n*	Solvent (%wt./V)	Water Detection Limit
4	Methanol (1)	1%
	Ethanol (2)	1%
	2-Propanol (5)	1%
6	Methanol (0.44)	1%
	Ethanol (5)	1%
	2-Propanol (5)	1%
10	Methanol (2)	1%
	Ethanol (5)	1%

## Data Availability

The original contributions presented in this study are included in the article/Supplementary Material. Further inquiries can be directed to the corresponding authors.

## Supplementary Materials

The following supporting information can be downloaded at: https://www.mdpi.com/article/10.3390/gels11010009/s1, Figure S1: ^1^H-NMR and signal assignment for the compound 3,6-THT-DiOH (6); Figure S2: ^1^H-NMR spectra of the studied 3,6-THTP-DiC*n*OH compounds; Figure S3: ^13^C-NMR spectra and signal assignment for the studied 3,6-THTP-DiC*n*OH compounds with *n* = 4, 6 and 10; Figure S4: Comparison of the diffractograms obtained for 3,6-THTP-DiC4OH in the crystal and LC phases.; Figure S5: Pictures of the gels obtained in methanol for the studied 3,6-THTP-DiC*n*OH compounds; Table S1: Description of the peak positions (2θ) of the XRD patterns obtained for 3,6-THT-DiC4OH and 3,6-THT-DiC6OH at different temperatures.
