# Supplementary material for: Columnar Mesophases and Organogels Formed by H-Bound Dimers Based on 3,6-Terminally Difunctionalized Triphenylenes
Source: Gels. 2024 Dec 27;11(1):9. doi: 10.3390/gels11010009 (PMC11764606; doi:10.3390/gels11010009)
Supplement: Supplementary file 1 [file gels-11-00009-s001.zip › gels-3351008-supplementary.pdf]

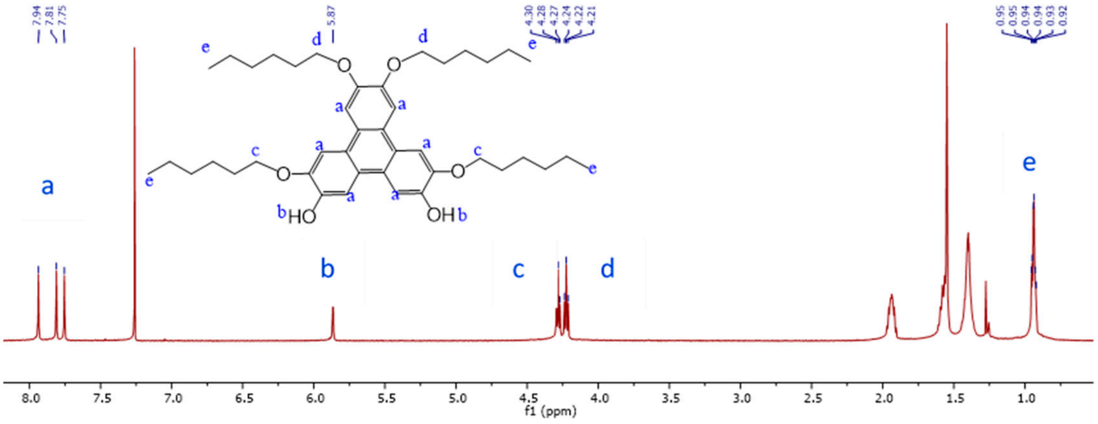


**Figure S1.** $^{1}$H-NMR and signal assignment for the compound 3,6-THT-DiOH (6).

 https://doi.org/10.3390/xxxxx

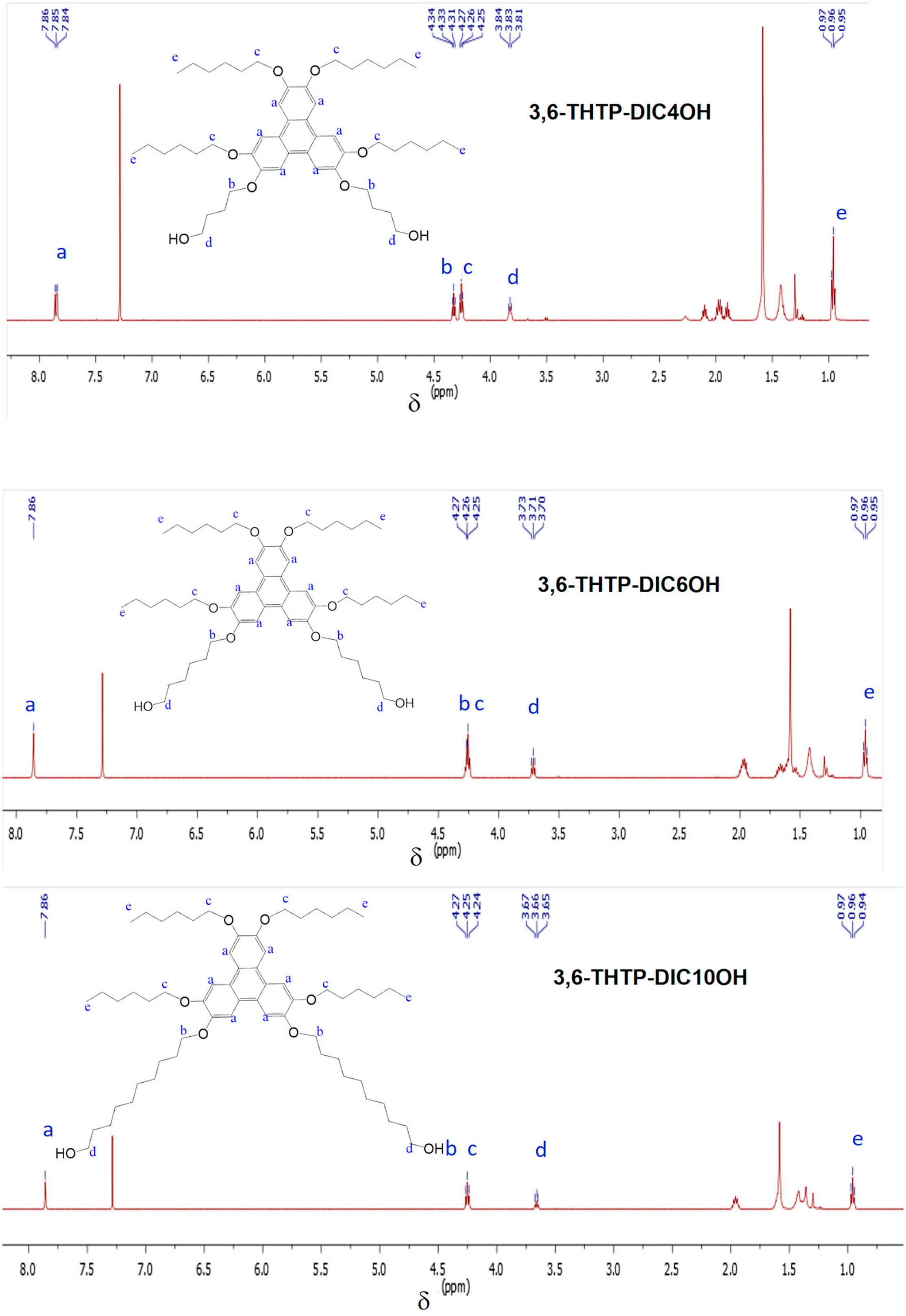


**Figure S2.** $^{1}$H-NMR spectra of the studied compounds

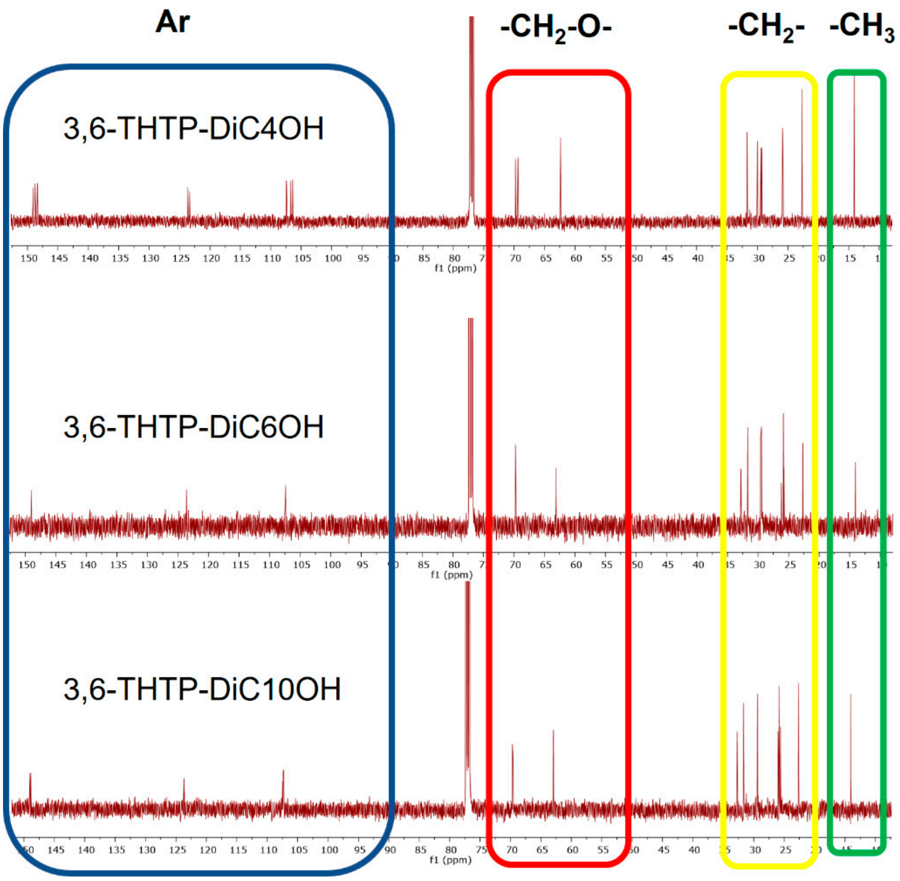


**Figure S3.** $^{13}$C-NMR spectra and signal assignment for the studied 3,6-THTP-DiCnOH compounds with *n* = 4 ,6 and 10.

**Table S1.** Description of the peak positions (2θ) of the XRD patterns obtained for 3,6-THT-DiC4OH and 3,6-THT-DiC6OH at different temperatures.

| 3,6 THTP-DiC*n*OH | Temperature | 2θ |
|---|---|---|
| *n* = 4 | 77 °C | 2.62; 5.23; 19 (wide); 25 |
| | 60 °C | 3.35: 5.03;6.68; 7.82; 10.18; 11.89; 13.10; 14.97; 15.46; 16.91; 18.15; 20.31; 21.13; 22.76; 24.26; 24.78; 26.15 |
| *n* = 6 | 60 °C | 2.51; 4.92; 19 (wide); 25 |
| | 49 °C | 2.72; 3.87; 4.5; 5.44; 6.17; 8.75; 9.87; 11.39; 12.30; 14.09; 14.87; 15.66; 16.04; 16.82; 17.68; 18.53; 19.84; 21.59; 22.94; 23.53; 24.78 |

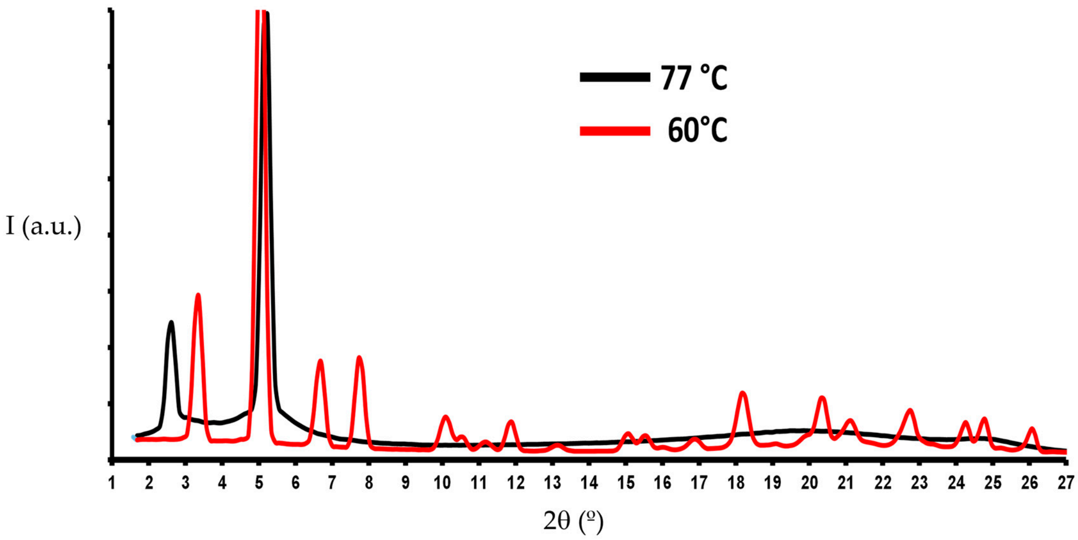


**Figure S4.** Comparison of the diffractograms obtained for 3,6-THTP-DiC4OH in the crystal and LC phases.

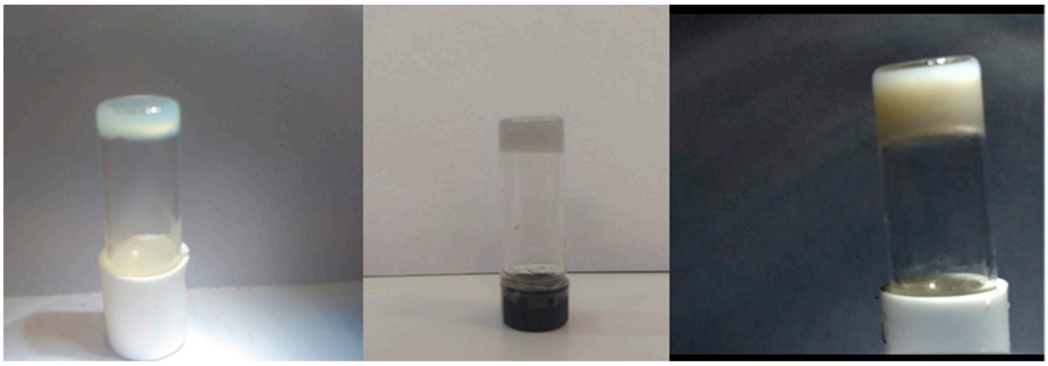

**Figure S5.** Pictures of the gels obtained in methanol for the studied 3,6-THTP-DiCnOH compounds (left: n = 4; middle: n = 6; right: n = 10).
